# Exposure to maternal cafeteria diets during the suckling period has greater effects on fat deposition and Sterol Regulatory Element Binding Protein-1c (SREBP-1c) gene expression in rodent offspring compared to exposure before birth

**DOI:** 10.1186/s12986-018-0253-3

**Published:** 2018-02-15

**Authors:** M. A. Vithayathil, J. R. Gugusheff, Z. Y. Ong, S. C. Langley-Evans, R. A. Gibson, B. S. Muhlhausler

**Affiliations:** 1 FOODplus Research Centre, Department of Wine and Food Sciences, School of Agriculture, Food and Wine, The University of Adelaide, Adelaide, South Australia 5064 Australia; 2 Healthy Mothers, Babies and Childrens Theme, South Australian Health and Medical Research Institute, Adelaide, South Australia 5001 Australia; 3 Sansom Institute for Health Research, University of South Australia, Adelaide, South Australia 5001 Australia; 4 School of Biosciences, University of Nottingham, Sutton Bonington, Loughborough, LE12 5RD UK

**Keywords:** Maternal nutrition, Cafeteria diet, Lactation, Pregnancy, Fat deposition, Fetal programming

## Abstract

**Background:**

While the adverse metabolic effects of exposure to obesogenic diets during both the prenatal and early postnatal period are well established, the relative impact of exposure during these separate developmental windows remains unclear. This study aimed to assess the relative contribution of exposure to a maternal cafeteria diet during pregnancy and lactation on body weight, fat mass and expression of lipogenic and adipokine genes in the offspring.

**Methods:**

Wistar rats were fed either a control chow (Control, *n* = 14) or obesogenic cafeteria diet (CAF, *n* = 12) during pregnancy and lactation. Pups were cross-fostered to another dam in either the same or different dietary group within 24 h of birth. Body weight, body fat mass and expression of lipogenic and adipokine genes in subcutaneous and visceral adipose tissues were determined in offspring at weaning and 3 weeks post-weaning.

**Results:**

Offspring suckled by CAF dams had a lower body weight (*P* < 0.05), but ~ 2-fold higher percentage body fat at weaning than offspring suckled by Control dams (*P < 0.01*), independent of whether they were born to a Control or CAF dam. At 6 weeks of age, after all offspring were weaned onto standard chow, males and females suckled by CAF dams remained lighter (*P* < 0.05) than offspring suckled by Control dams, but the percentage fat mass was no longer different between groups. Sterol Regulatory Element Binding Protein-1c (SREBP-1c) mRNA expression was ~ 25% lower in offspring suckled by cafeteria dams in males at weaning (*P* < 0.05) and in females at 6 weeks of age (*P* < 0.05). Exposure to a cafeteria diet during the suckling period alone also resulted in increased adipocyte Peroxisome Proliferator Activated Receptor-γ (PPAR-γ) mRNA expression in females, and adiponectin and leptin mRNA expression in both sexes at weaning.

**Conclusions:**

The findings from this study point to the critical role of the suckling period for deposition of adipose tissue in rodents, and the potential role of altered adipocyte gene expression in mediating these effects.

## Background

Maternal obesity and consumption of obesogenic diets during pregnancy and lactation has long been shown to heighten the risk of obesity and type 2 diabetes in the offspring [1, 2]. This effect is thought to be due to exposure of the developing fetus/neonate to an increased nutrient supply during critical periods of development, which results in permanent alterations to the structure, gene expression profile and function of key organs and regulatory systems responsible for metabolic control [3].

While the adverse metabolic effects of exposure to obesogenic diets during both the prenatal and early postnatal periods are well established, the relative impact of exposure during these separate developmental periods remains unclear [4–6]. This has particular relevance for developing strategies for intervention, since it informs whether the adverse effects of prenatal exposures could be reversed by interventions applied in the early postnatal period. This question cannot be readily addressed in human studies, and the few existing experimental animal studies have produced conflicting results. Sun and colleagues reported that rat pups cross-fostered onto dams consuming a high-fat diet had a higher percentage body fat at 3 weeks of age than pups who were suckled by a control dam, independent of the diet they had been exposed to before birth [5]. In contrast, Chang and colleagues showed that rat offspring born to dams given a high-fat diet had a higher fat mass in young adulthood than those born to control dams, independent of their diet after birth [6]. Given these contrasting reports, there is a need for further investigations to clearly identify the importance of the timing of high-fat diet exposure on the offspring. The existing literature in this area is challenging to interpret as it has been impossible to assess whether the observed phenotypes in the offspring are a consequence of maternal obesity or of other aspects of the obesity-inducing diets, as these tend to be high in single fat sources. Cafeteria feeding provides a useful alternative to the feeding of purified high-fat diets to induce obesity. Feeding a diet of high variety and novelty induces persistent hyperphagia and is a better representation of human obesogenic diets [7, 8].

Programming of the adipogenic and/or lipogenic capacity of the developing fat cell, or adipocyte, has been shown to be a key mechanism underlying the programming of obesity following an altered perinatal nutritional environment [9]. Existing evidence from studies in both humans and animal models shows that relatively minor perturbations in early adipose tissue development may increase the risk of overweight and obesity later in life [10, 11]. The capacity of pre-adipocytes in humans to proliferate and differentiate during adult life is much lower than before birth and in early infancy and most, if not all, adipose tissue development is completed by 12 months of age [12]. Thus, the fetal and early postnatal period are critical windows in the development of adipose depots, and this process is highly sensitive to the nutritional environment an individual experiences during this time [12, 13]. Previous studies have demonstrated that the offspring of rat dams fed a cafeteria diet during pregnancy and lactation exhibit increased adipocyte proliferation of adipose cells and increased expression of adipogenic and lipogenic genes in adulthood [11]. However, the relative importance of exposure to an increased nutrient supply before birth and during the early postnatal period for the programming of altered adipocyte gene expression is yet to be determined.

The aim of the current study was to utilise a cross-fostering paradigm in a rodent model to assess the relative contribution of exposure to a maternal cafeteria diet before birth and during the suckling period on body weight, fat mass and expression of key lipogenic and adipokine genes in the offspring at weaning and in early adolescence after weaning onto a control diet.

## Methods

### Animals and feeding regimen

This study was approved by the Animal Ethics Committee of the University of Adelaide (Approval Number: S_2010_034). Twenty-six female Albino Wistar rats (200-250 g) and four male Albino Wistar rats (200-300 g) were used in this study. All rats were individually housed under a 12 h light/12 h dark cycle at a room temperature of 25 °C and allowed to acclimatise to the animal housing facility for at least 1 week before initiation of the experiment. During this time rats were fed ad libitum on standard rodent feed (Specialty Feeds, Glen Forrest, Western Australia, Australia, 25 g/day) with free access to water.

At the end of the acclimatisation period, rats were randomly assigned to either the Control (*n* = 14) or a Cafeteria (CAF; *n* = 12) group. Control rats were given free access to standard rodent feed while CAF rats were fed a high-fat/high-sugar cafeteria diet. The cafeteria diet comprised of 10 g peanut butter, 10 g hazelnut spread, 7 g chocolate-flavoured biscuits (4 biscuits), 10 g extruded savoury snacks, 2.5 g sweetened multi-grain breakfast cereal and 50 g of a 15%lard/85%rodent feed mix provided every 2 days [14]. The macronutrient composition of the dietary components is shown in Table 1. Individual food intake was determined in all dams every 2 days before pregnancy, during pregnancy and lactation, uneaten food items removed and fresh food provided. All female rats were weighed once per week throughout the experiment.

**Table 1:** Nutritional details of the individual components of the cafeteria diet and standard rat chow

	Energy (kJ/g)	Fat (%)	Carbohydrates (%)	Protein (%)	Sodium (mg/g)
Lard/ Rodent Feed mixture	20.9	19	51	17	3
Peanut Butter	26.2	50	25	21	6
Hazelnut Spread	23.3	36	54	4	1
Chocolate-Flavoured Biscuits	20.0	15	71	10	3
Savoury Extruded Snacks	23.1	35	51	7	10
Sweetened Breakfast Cereal	27.3	3	43	1	1
Standard Rodent Feed	18.0	5	60	20	4

### Mating and pregnancy

After 4 to 6 weeks on their respective diets, vaginal smears were conducted daily on all females to determine their stage in the estrous cycle. On the evening of diestrous/proestrous, two female rats were placed in a group cage with a male rat for 24 h. Vaginal smears were performed the following morning to check for the presence of sperm in order to confirm successful mating and this was designated as gestation day 0. Female rats were then removed from the males and housed individually thereafter. Female rats were maintained on the same diet as before mating throughout pregnancy and lactation. Pregnancy outcomes (gestation length, litter size, number of dead and live pups and number of male and female pups) were recorded for all dams within 24 h of birth.

### Cross-fostering

**Fig. 1:**
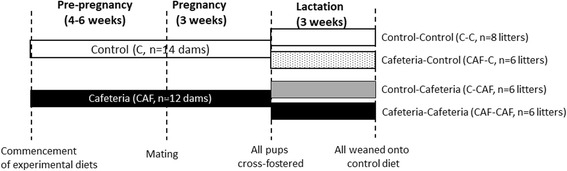
Diagram of experimental design

All dams were allowed to give birth naturally and all pups were born on day 21–22 of gestation. Within 24 h of birth, all litters were culled to 8 pups, with 4 males and 4 females where possible in order to standardise litter size for suckling. Pups were then cross-fostered to another dam that gave birth within the same 24 h period from either the same or different dietary treatment group. This resulted in 4 groups of offspring: litters from a Control dam cross-fostered onto another Control dam (Control-Control, C-C, *n* = 8), litters from a Control dam cross-fostered onto a CAF dam (Control-CAF, C-CAF, *n* = 6), litters from a CAF dam cross-fostered onto a Control dam (CAF-Control, CAF-C, *n* = 6) and litters from a CAF dam cross-fostered onto another CAF dam (CAF-CAF, *n* = 6) (Fig. 1).

Pups remained with their foster mothers until weaning (3 weeks of age). After weaning, the pups were housed with their same-sex littermates (3–4 pups/cage) and were fed with standard rat chow (Specialty Feeds, Glen Forrest, Western Australia, Australia) until 6 weeks of age. Pups were weighed every second day until weaning and once per week thereafter until the end of the experiment.

### Intraperitoneal glucose tolerance tests

Intraperitoneal glucose tolerance tests (IPGTT) were performed on 1 male and 1 female pup per litter after an overnight fast of ~ 18 h at 6 weeks of age. Baseline blood samples were collected from the tail vein and a glucose bolus (2 g/kg of 50% dextrose in sterile 0.9% saline) was then injected intraperitoneally. Blood samples were drawn from the tail vein at 5, 10, 15, 30, 60 and 120 min post glucose infusion. Blood glucose concentrations were tested on a calibrated handheld glucometer (Accu-Chek Performa©, Roche, Germany) using test reagent strips. Tests were performed in duplicate at each time point. The trapezoidal rule was used to determine the incremental area under the glucose curve (AUC) for all experimental animals.

### Post-mortem and tissue collection

One male and one female pup from each litter were killed at weaning (3 weeks of age) and at 6 weeks of age. All rats were weighed immediately prior to post-mortem and were then killed with an overdose of CO_2_. The rats were not fasted and all post-mortems were conducted in the light phase between 8 and 10 am. Nose-to-anus length and abdominal circumference were recorded to the nearest millimetre. Blood samples were collected by cardiac puncture, and blood was centrifuged at 3500 g, 4 °C for 15 min and plasma stored at − 20 °C for subsequent analysis of hormone and metabolite concentrations. Organs and individual fat depots including retroperitoneal fat, omental fat, gonadal fat, interscapular fat and subcutaneous fat were dissected and weighed. The weight of all organs was recorded and a sample of retroperitoneal (visceral) and subcutaneous adipose tissue from each pup was snap-frozen in liquid nitrogen and stored at − 80 °C for subsequent gene expression analyses.

### Determination of hormone and metabolite concentrations

Plasma concentrations of glucose and non-esterified fatty acids (NEFA) were determined using the Infinity Glucose Hexokinase kit (Thermo Electron, Pittsburgh, PA, USA) and the Wako NEFA C kit (Wako Pure Chemical Industries Ltd., Osaka, Japan), respectively. Assays were conducted using a Konelab 20 (Thermo Scientific, Vantaa, Finland). Plasma insulin and leptin concentrations were measured by immunoassay using the ALPCO Insulin (Rat) Ultrasensitive ELISA kit (ALPCO diagnostics, Salem, NH, USA) and the Crystal Chem Rat Leptin ELISA kit (Crystal Chem INC, Downers Grove, IL, USA). All assays were conducted according to manufacturer’s instructions and intra- and inter-assay coefficients of variation were < 10%.

### Determination of gene expression in the subcutaneous and retroperitoneal adipose tissue

Total mRNA was extracted from samples of ~ 100 mg of retroperitoneal (visceral) adipose tissue and ~ 100 mg of subcutaneous adipose tissue from each pup using Trizol reagent (Invitrogen Australia, Mount Waverley, Vic, Australia). The RNA was then purified using an RNeasy Mini kit (Qiagen Australia, Doncaster, Vic, Australia) and cDNA synthesized using Superscript III reverse transcriptase (Invitrogen Australia) and random hexamers.

The relative expression of PPAR-γ, SREBP-1c, Fatty Acid Synthase (FAS), Glycerol-3-Phosphate Dehydrogenase (G3PDH), leptin and adiponectin mRNA transcripts was determined by Quantitative real time PCR (qRT PCR) using the SYBR green system on the Applied Biosystems ViiA 7 Real-Time PCR machine (Applied Biosystems, Foster City, CA, USA). All primers had been validated previously for use in rat tissues [15]. All primers were sequenced prior to the experiment to ensure the authenticity of the DNA product and a qRT-PCR melt curve analysis was performed to demonstrate amplicon homogeneity. Primer sequences are shown in Table 2 and mRNA expression of the reference gene β-actin was measured using the β-actin Quantitect primer assay (Qiagen Australia, Doncaster, Vic, Australia). The abundance of each mRNA transcript was quantified relative to the two housekeeper genes (β-actin and cyclophilin Pα, CYPα) using the Applied Biosystems Data Assist software (Applied biosystems, Foster City, CA, USA). Three quality controls as well as two negative controls for each primer were included on each 96-well plate in order to verify inter-plate consistency, and the inter-plate CoV was < 5% for all experiments.

**Table 2:** Primers sequences used for the determination of gene expression in adipose tissue by qRT-PCR

Gene	Forward Primer (5′-3′)	Reverse Primer (5′-3′)	Accession No.
PPAR-γ	TCCTCCTGTTGACCCAGAGCAT	AGCTGATTCCGAAGTTGGTGG	NM_013124
SREBP-1c	TGCGGACGCAGTCTGGGCAAC	GTCACTGTCTTGGTTGTTGATG	AF_286469
FAS	TGCTCCCAGCTGCAGGC	GCCCGGTAGCTCTGGGTGTA	NM_017332
G3PDH	GCTTCGGTGACAACACCA	AGCTGCTCAATGGACTTTCC	NM_022215
Adiponectin	AATCCTGCCCAGTCATGAAG	CATCTCCTGGGTCACCCTTA	NM_144744
Leptin	ATTTCACACACGCAGTCGGTATCCG	CCAGCAGATGGAGGAGGTC	NM_013076
CYPα	TATCTGCACTGCCAAGACTGAGTG	CTTCTTGCTGGTCTTGCCATTCC	NM_017101

### Statistics

#### Justification of sample size

The sample size for this study was calculated based on fat mass in the offspring at weaning and at 6 weeks of age as the primary outcomes. Based on our previous studies we expected the mean value for fat mass in the control group of 1.5% of body weight at weaning and 1.6% at 3 months of age with a standard deviation of 0.30% at both ages. Thus, an *n* = 6 litters/dams per group provided a power of 0.9 at an alpha of 5% (*P* < 0.05) to detect a biologically meaningful difference of 0.5% body fat mass (a conservative estimate) at both time points. Sample size calculations were performed in consultation with a statistician.

#### Statistical analyses

Data are presented as mean ± SEM. The effect of maternal cafeteria diet on maternal body weight, maternal intake of total energy, fat and carbohydrate before pregnancy, during pregnancy and lactation was determined using a Student’s unpaired t-test. The effect of the cafeteria diet on pregnancy outcomes were similarly determined, with the exception of the proportion of dead and live pups, which was compared between groups using a Chi-squared test. The effect of maternal diet and sex on offspring body weight, body fat mass, plasma leptin concentrations and expression of lipogenic genes at 3 and 6 weeks of age were determined using a three-way ANOVA, with sex, prenatal and postnatal diet as factors. Due to differences in variance between male and female offspring for a number of measures, as determined by the three-way ANOVA, differences between groups were assessed separately in male and female offspring using a 2-way ANOVA. If a significant interaction between prenatal and postnatal dietary exposure was determined, the effect of diet during each period was analysed separately by Student’s t-test. All analyses were performed using SPSS for Windows Version 24.0 (SPSS Inc., Chicago, IL, USA). A probability of *P* < 0.05 was considered statistically significant in all analyses.

## Results

### Maternal and pregnancy outcomes

Data on food and nutrient intakes of the animals has been previously published [4]. Energy intake (kJ per day) was not significantly different between the CAF and Control dams before pregnancy (Control 369 ± 32 kJ/ d, CAF 426 ± 8 kJ/d), but was significantly higher in CAF dams during pregnancy (Control 447 ± 11 kJ/d, CAF 501 ± 12 kJ/d, *P* < 0.01) and lower during lactation compared to Control dams (Control 2643.8 ± 142.6 kJ/g/d, CAF 2001.6 ± 82.1 kJ/g/d, *P* < 0.01). Dams fed the cafeteria diet consumed more fat (pregnancy: Control 3.2 ± 0.2 g/kg/d, CAF 15.3 ± 0.7 g/kg/d, *P* < 0.01; lactation: Control 6.8 ± 0.4 g/kg/d, CAF 26.4 ± 1.4 g/kg/d, *P* < 0.01) but less protein (pregnancy; Control 13.5 ± 0.8 g/kg/d, CAF 6.6 ± 0.2 g/kg/d, *P* < 0.01; lactation: Control 28.8 ± 1.6 g/kg/d, CAF 12.4 ± 0.6 g/kg/d, *P* < 0.01) and carbohydrate (pregnancy, Control 41.4 ± 2.4 g/kg/d, CAF 29.6 ± 1.5 g/kg/d, *P* < 0.01; lactation: Control 88.8 ± 4.8 g/kg/d, CAF 49.9 ± 1.8 g/kg/d, *P* < 0.01) compared to Control dams throughout the feeding period [4]. There was no difference in the body weight of the Control and CAF dams before the cafeteria diet was introduced (Control = 257 ± 11.6 g, CAF = 268 ± 17.6 g), but CAF dams were ~ 20% heavier than Control dams at mating (Control = 315 ± 6.0 g, CAF = 393 ± 8.6 g, *P* < 0.01), immediately after delivery (Control = 363.38 ± 7.2 g, CAF = 450.03 ± 9.5 g, *P* < 0.01) and at the end of lactation (Control = 360.71 ± 4.1 g, CAF = 427.86 ± 7.4 g, *P* < 0.01).

There were no differences between Control and CAF dams in gestation length, litter size or the percentage of male and female pups, however body weights of pups at birth were ~ 20% lower in the CAF group (Table 3). There were also significantly more litters in which pups died either before or shortly after birth in the CAF group (Table 3).

**Table 3:** Pregnancy and birth outcomes in Control and CAF dams

	Control dams (*n* = 14) Mean	SEM	CAF dams (*n* = 12) Mean	SEM
Gestational age (days)	22	0.10	22	0.001
Litter size	13	0.65	13	0.68
Birth weight (g)	7.15	0.17	6.03^**^	0.13
Live pups	13	0.77	12	0.65
Litters with dead pups (N,%)^a^	0,0%		5,42%^**^	
Percent male pups	47.7	2.6	55.20	3.7

Values are expressed as mean ± SEM. Differences between groups assessed by a Student’s unpaired test

^**^denotes significance at *P* < 0.01

^a^Values expressed as percentage of total litters. Differences between groups assessed by Chi-squared test

### Offspring outcomes

#### Bodyweight, length and abdominal circumference

**Fig. 2:**
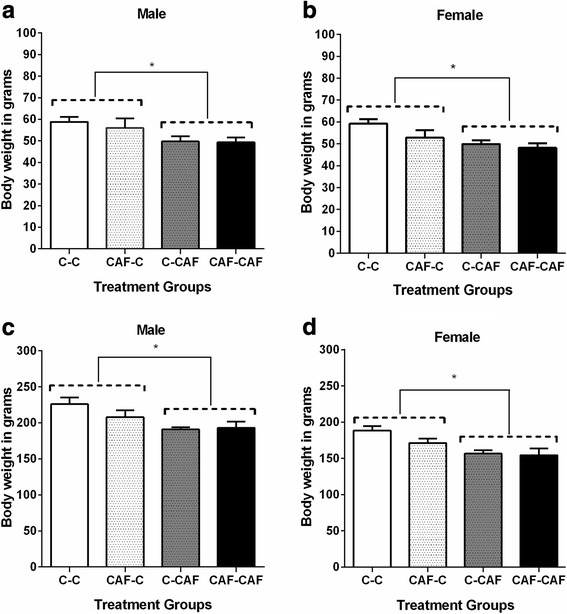
Body weight at weaning (**a**, **b**) and at 6 weeks of age (**c**, **d**) in male and female offspring exposed to a maternal cafeteria diet during the prenatal and/or suckling period. Values are expressed as mean ± SEM. * denotes significant differences between treatment groups *(P < 0.05) (3 weeks: C-C, n = 9, CAF-C, n = 5; C-CAF, n = 6 females, 4 males; CAF-CAF: n = 8; 6 weeks: C-C, n = 9, CAF-C, n = 6 females, 4 males; C-CAF n = 6 females, 4 males; CAF-CAF, n = 7)*

At weaning (3 weeks of age), both male and female offspring that were cross-fostered to CAF dams were ~ 15% lighter and ~ 10% shorter compared to those suckled by Control dams (Fig. 2a, b; Table 4). The abdominal circumference at weaning was also ~ 7% lower in offspring suckled by a CAF dam in females, but not different between groups in male offspring (Table 4). All effects were independent of whether offspring had been born to a control or CAF dam.

**Table 4:** Length, abdominal circumference and individual fat depots as a percentage of bodyweight in male and female offspring at 3 and 6 weeks of age

	Male								Female							
	C-C *n* = 9		CAF-C *n* = 5		C-CAF *n* = 4		CAF-CAF *n* = 7		C-C *n* = 9		CAF-C *n* = 6		C-CAF *n* = 6		CAF-CAF *n* = 7	
	Mean	SEM	Mean	SEM	Mean	SEM	Mean	SEM	Mean	SEM	Mean	SEM	Mean	SEM	Mean	SEM
3 weeks																
Nose-tail length (cm)	14.90^b^	0.20	14.50^b^	0.50	13.50^a^	0.10	13.40^a^	0.30	14.30^b^	0.30	14.20^b^	0.40	13.20^a^	0.30	13.30^a^	0.20
Abcirc (cm)	10.70	0.30	10.30	0.10	10.20	0.20	10.40	0.20	10.60^b^	0.10	10.60^b^	0.30	9.90^a^	0.40	9.80^a^	0.20
Gonadal fat	0.19^a^	0.02	0.20^a^	0.02	0.37^b^	0.05	0.38^b^	0.04	0.27^a^	0.03	0.24^a^	0.03	0.61^b^	0.05	0.61^b^	0.06
Interscapular fat	0.63^a^	0.03	0.59^a^	0.04	1.04^b^	0.11	0.86^b^	0.1	0.64^a^	0.04	0.69^a^	0.03	0.90^b^	0.04	0.84^b^	0.04
Retroperitoneal	0.40^a^	0.02	0.40^a^	0.07	0.76^b^	0.05	0.96^b^	0.07	0.43^a^	0.02	0.33^a^	0.02	0.75^b^	0.05	0.72^b^	0.02
Omental fat	0.55^a^	0.03	0.51^a^	0.06	0.77^b^	0.06	0.73^b^	0.05	0.48^a^	0.03	0.55^a^	0.04	0.65^b^	0.02	0.78^b^	0.04
Subcutaneous fat	3.88^a^	0.18	4.66^a^	0.35	8.22^b^	0.58	9.27^b^	0.57	4.92^a^	0.36	4.55^a^	0.49	9.36^b^	0.23	8.57^b^	0.47
6 weeks																
Nose-tail length (cm)	22.10^b^	0.30	21.10^a^	0.30	21.80^b^	0.10	21.10^a^	0.30	21.20^b^	0.30	20.60^b^	0.30	20.00^a^	0.30	19.80^a^	0.40
Abcirc (cm)	16.50	0.40	16.90	0.50	16.20	0.20	16.00	0.30	15.50^b^	0.30	15.10^b^	0.50	14.80^a^	0.30	14.40^a^	0.30
Gonadal fat	0.67	0.05	0.74	0.10	0.72	0.04	0.69	0.02	0.97	0.07	0.80	0.13	0.77	0.08	0.77	0.12
Interscapular fat	0.31	0.03	0.34	0.05	0.39	0.03	0.36	0.02	0.34^a^	0.02	0.31^a^	0.01	0.37^b^	0.03	0.38^b^	0.02
Retroperitoneal	0.74	0.09	0.87	0.08	0.86	0.06	0.87	0.06	0.77	0.06	0.64	0.05	0.72	0.06	0.80	0.11
Omental fat	0.71	0.05	0.68	0.04	0.74	0.03	0.74	0.07	0.84	0.03	0.64	0.07	0.69	0.05	0.68	0.07
Subcutaneous fat	4.08	0.24	4.88	0.21	4.02	1.09	4.63	0.14	4.63	0.28	3.73	0.36	4.48	0.36	4.31	0.18

Values are expressed as means ± SEM. Differences between groups and sexes assessed by 3-way ANOVA. Different superscripts indicate significant differences between groups (*P* < 0.05)

Offspring suckled by CAF dams remained lighter than the offspring suckled by Control dams after weaning, and were still ~ 25 g (14%) lighter at 6 weeks of age in both males and females, independent of the prenatal nutritional environment (Fig. 2c, d). Female offspring suckled by CAF dams also remained shorter and had a lower abdominal circumference than those suckled by Control dams, although this effect was less pronounced than at weaning (Table 4).

#### Body fat mass

**Fig. 3:**
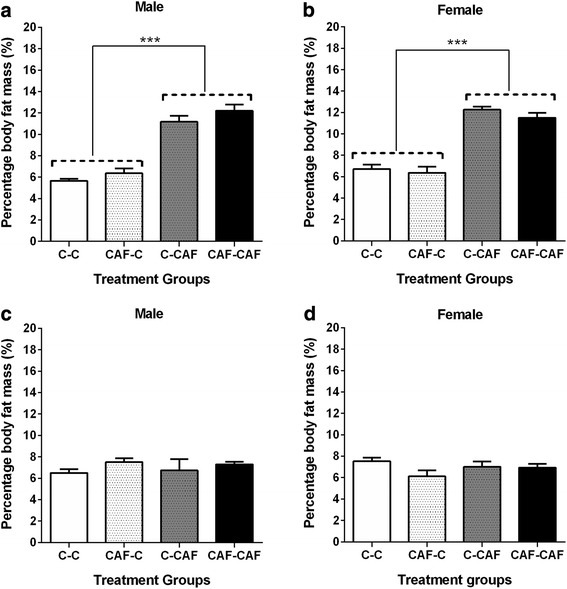
Percentage body fat mass at weaning (**a**, **b**) and at 6 weeks of age (**c**, **d**) in male and female offspring exposed to a maternal cafeteria diet during the prenatal and/or suckling period. Values are expressed as mean ± SEM. *** denotes significant differences between treatment groups *(P < 0.01) (3 weeks: C-C, n = 9 males, 9 females; CAF-C, n = 5 males, 5 females; C-CAF, n = 4 males, 6 females; CAF-CAF: n = 8 males, 8 females; 6 weeks: C-C, n = 9 males, 9 females; CAF-C, n = 4 males, 6 females; C-CAF n = 4 males, 6 females; CAF-CAF, n = 7 males, 7 females)*

The percentage total body fat mass at weaning was ~ 2-fold higher in both male and female offspring suckled by CAF compared to Control dams, independent of whether they were born to a Control or CAF dam (*P* < 0.05, Fig. 3a, b). The relative mass of individual fat depots including that gonadal fat, interscapular fat, retroperitoneal fat, omental fat and subcutaneous fat were also all between ~ 1.5 and 2-fold higher in offspring suckled by a CAF dam compared to offspring suckled by a Control dam in both males and females (Table 4).

At 6 weeks there was no longer any difference in percentage total body fat mass between any of the treatment groups in either males or females (Fig. 3c, d). In females, but not in males, the mass of the interscapular fat depot as a percentage of body weight was higher in offspring suckled by CAF dams compared to offspring suckled by Control dams, independent of whether they were born to a Control or CAF dam, however the difference was relatively modest (1.15 fold) (Table 4). There were no differences between groups in the relative weight of other fat depots.

#### Glucose tolerance

There were no significant differences in glucose tolerance at 6 weeks of age, as assessed by the Glucose AUC during the IPGTT, in either male or female offspring (Male glucose_AUC_: C-C = 1140.2 ± 56.8, CAF-C = 1155.8 ± 58.3, C-CAF = 1227.4 ± 57.7, CAF-CAF = 1025.9 ± 116.0; Female glucose_AUC_: C-C = 1111.0 ± 37.1, CAF-C = 1155.0 ± 98.9, C-CAF = 1201.2 ± 42.1, CAF-CAF = 1122.9 ± 67.1). Fasting glucose concentrations at baseline, and the peak glucose concentrations attained during the IPGTT were also not different between treatment groups (data not shown).

#### Plasma hormone and metabolite concentrations

Female offspring born to CAF dams had non-fasting plasma glucose concentrations at 3 weeks of age that were ~ 2.5 mmol/l and ~ 20% higher than offspring born to Control dams, independent of their nutritional exposure during the suckling period (Table 5). Plasma glucose, insulin and NEFA concentrations were not different between groups at 6 weeks of age in either males or females (Table 5).

**Table 5:** Plasma concentrations of glucose, NEFA, insulin^ƚ^ and leptin in male and female offspring at 3 and 6 weeks of age

	Male								Female							
	C-C *n* = 9		CAF-C *n* = 5		C-CAF *n* = 4		CAF-CAF *n* = 6		C-C *n* = 9		CAF-C *n* = 6		C-CAF *n* = 6		CAF-CAF *n* = 7	
	Mean	SEM	Mean	SEM	Mean	SEM	Mean	SEM	Mean	SEM	Mean	SEM	Mean	SEM	Mean	SEM
3 weeks																
Glucose (mmol/l)	13.01	1.34	13.68	0.91	14.23	1.79	14.35	2.00	10.02^a^	0.51	13.35^b^	1.28	12.15^a^	1.04	13.71^b^	1.28
NEFA (mEq/l)	0.47	0.10	0.57	0.09	0.59	0.07	0.46	0.11	0.45	0.10	0.65	0.10	0.65	0.15	0.63	0.08
Leptin (ng/ml)	5.68^a^	0.87	5.84^a^	1.85	7.85^b^	2.40	15.38^b^	1.59	5.84^a^	0.74	6.49^a^	1.39	10.56^b^	1.92	13.77^b^	1.81
6 weeks																
Glucose (mmol/l)	17.71	1.50	15.15	1.78	16.91	2.88	13.77	0.93	18.31	2.55	13.49	0.44	14.59	1.12	14.57	0.98
NEFA (mEq/l)	0.61	0.07	0.53	0.07	0.60	0.04	0.65	0.12	0.73	0.05	0.51	0.08	0.63	0.1	0.69	0.10
Insulin (ng/ml)	0.98	0.29	0.23	0.01	0.37	0.29	0.89	0.52	1.05	0.32	0.72	0.37	0.44	0.07	1.49	0.54
Leptin (ng/ml)	5.04	0.47	5.97	0.87	6.05	0.72	5.78	0.43	5.03^b^	0.36	3.52^a^	0.51	5.14^b^	0.44	4.20^a^	0.49

^ƚ^insulin could not be assessed at 3 weeks of age. Values are expressed as means ± SEM. Differences between groups and sexes assessed by 3-way ANOVA. Different superscripts denote significant differences between groups (*P* < 0.05)

At 3 weeks of age, plasma leptin concentrations were 2-fold higher in both males and female offspring suckled by CAF dams compared to offspring suckled by Control dams, independent of their prenatal nutritional exposure (Table 5). When data from all treatment groups were combined, circulating plasma leptin concentrations were positively correlated both to leptin mRNA expression in the subcutaneous adipose tissue (male: *r*^*2*^ = 0.40, *P* < 0.01; female: *r*^*2*^ = 0.55, *P* < 0.001) and to relative subcutaneous fat mass (male: *r*^*2*^ = 0.58, *P* < 0.001, female: *r*^*2*^ = 0.38, *P* < 0.01) in both male and female offspring.

There were no differences in plasma leptin concentrations between groups in male offspring at 6 weeks of age (Table 5). At 6 weeks of age, female offspring born to CAF dams had slightly (~ 9%) lower plasma leptin concentrations compared to female offspring born to Control dams, independent of whether they were suckled by a Control or CAF dam (Table 5).

#### PPAR-γ, SREBP-1c, FAS and G3PDH gene expression in visceral and subcutaneous adipose tissue

At 3 weeks of age, female offspring suckled by CAF dams had a ~ 20% higher relative expression of PPAR-γ mRNA in the subcutaneous adipose tissue compared to those suckled by a Control dam (*P* < 0.05, Table 6). This effect was not observed in male offspring. There was no effect of either prenatal or postnatal exposure to the cafeteria diet on PPAR-γ mRNA expression in retroperitoneal adipose tissue in either male or female offspring at this time point. At 6 weeks of age, when all offspring had been fed a standard chow diet for 3 weeks after weaning, PPAR-γ mRNA expression in retroperitoneal fat was ~ 60% lower in female offspring who had been born to CAF dam but suckled by a Control dam compared to those pups both born to and suckled by a Control dam (Table 6). There were no differences between groups in PPAR-γ mRNA expression in retroperitoneal adipose tissue in males or in the subcutaneous adipose tissue in either male or in female offspring at 6 weeks of age (Table 7).

**Table 6:** Mean normalised expression of lipogenic and adipokine genes in subcutaneous and retroperitoneal adipose tissue in male and female offspring at 3 weeks of age

	Male								Female							
	C-C *n* = 7		CAF-C *n* = 4		C-CAF *n* = 4		CAF-CAF *n* = 76		C-C *n* = 7		CAF-C *n* = 4		C-CAF *n* = 5		CAF-CAF *n* = 7	
	Mean	SEM	Mean	SEM	Mean	SEM	Mean	SEM	Mean	SEM	Mean	SEM	Mean	SEM	Mean	SEM
Subcutaneous																
PPAR-γ	0.06	0.01	0.07	0.01	0.08	0.00	0.07	0.01	0.06^a^	0.01	0.06^a^	0.01	0.07^b^	0.01	0.08^b^	0.01
FAS	0.94	0.26	0.56	0.16	0.77	0.54	0.44	0.10	0.69	0.10	0.77	0.23	0.85	0.24	0.54	0.10
G3PDH	1.61	0.31	1.11	0.16	1.26	0.28	1.39	0.36	1.58	0.32	1.65	0.06	1.70	0.03	1.82	0.20
Adiponectin	0.68	0.11	0.96	0.05	1.40	0.37	0.92	0.14	0.76^a^	0.08	0.87^a^	0.10	1.62^b^	0.16	1.29^b^	0.18
Leptin	0.08^a^	0.02	0.10^a^	0.02	0.18^b^	0.02	0.19^b^	0.03	0.12^a^	0.03	0.12^a^	0.03	0.22^b^	0.02	0.27^b^	0.03
Retroperitoneal																
PPAR-γ	0.07	0.01	0.08	0.02	0.09	0.02	0.06	0.00	0.05	0.01	0.06	0.02	0.04	0.01	0.06	0.00
FAS	1.44	0.24	0.94	0.16	1.02	0.34	0.77	0.19	1.40	0.22	1.32	0.43	1.16	0.04	1.02	0.22
G3PDH	1.25	0.18	0.93	0.33	1.00	0.09	1.78	0.42	1.05	0.18	1.06	0.40	1.37	0.31	1.58	0.23
Adiponectin	0.73^a^	0.09	0.95^a^	0.32	2.26^b^	0.28	2.27^b^	0.71	0.59^a^	0.09	0.65^a^	0.16	0.88^b^	0.10	1.13^b^	0.17
Leptin	0.08^a^	0.01	0.06^a^	0.01	0.21^b^	0.06	0.21^b^	0.07	0.08^a^	0.02	0.06^a^	0.02	0.09^b^	0.02	0.20^b^	0.04

Values are expressed as means ± SEM. Differences between groups and sexes assessed by 3-way ANOVA. Different superscripts denote significant differences between groups (*P* < 0.05)

**Table 7:** Mean normalised expression of lipogenic and adipokine genes in subcutaneous and retroperitoneal adipose tissue in male and female offspring at 6 weeks of age

	Male								Female							
	C-C *n* = 8		CAF-C *n* = 4		C-CAF *n* = 4		CAF-CAF *n* = 6		C-C *n* = 9		CAF-C *n* = 6		C-CAF *n* = 5		CAF-CAF *n* = 6	
	Mean	SEM	Mean	SEM	Mean	SEM	Mean	SEM	Mean	SEM	Mean	SEM	Mean	SEM	Mean	SEM
Subcutaneous																
PPAR-γ	0.05	0.01	0.05	0.01	0.04	0.01	0.05	0.01	0.03	0.00	0.04	0.01	0.02	0.01	0.03	0.01
FAS	0.58	0.10	0.49	0.10	0.83	0.26	0.73	0.13	0.60	0.04	0.30	0.05	0.24	0.06	0.42	0.15
G3PDH	0.42	0.06	0.37	0.08	0.37	0.13	0.51	0.09	0.26	0.06	0.21	0.03	0.21	0.08	0.25	0.06
Adiponectin	1.62	0.27	1.44	0.37	1.75	0.78	2.35	0.30	1.10	0.22	1.13	0.28	0.72	0.19	1.00	0.14
Leptin	0.12	0.03	0.09	0.03	0.11	0.05	0.20	0.05	0.08	0.02	0.08	0.02	0.05	0.02	0.08	0.01
Retroperitoneal																
PPAR-γ	0.13	0.03	0.19	0.03	0.13	0.03	0.13	0.02	0.16^b^	0.01	0.10^a^	0.01	0.14^ab^	0.01	0.14^ab^	0.01
FAS	2.21^a^	0.48	4.24^b^	0.60	3.30^ab^	0.58	2.82^ab^	0.23	2.85	0.28	2.26	0.50	2.67	0.41	2.63	0.39
G3PDH	1.31^a^	0.28	2.80^b^	0.45	2.13^ab^	0.35	1.95^ab^	0.24	1.72	0.18	1.19	0.22	1.70	0.23	1.64	0.21
Adiponectin	1.62	0.43	1.86	0.39	2.64	0.07	1.72	0.25	1.86^b^	0.30	0.90^a^	0.13	1.49^b^	0.18	1.14^a^	0.08
Leptin	0.10	0.03	0.15	0.07	0.22	0.07	0.13	0.01	0.20^b^	0.04	0.05^a^	0.01	0.14^b^	0.04	0.10^a^	0.01

Values expressed as mean ± SEM, *n* = 4–6 for all groups. Differences between groups and sexes assessed by 3-way ANOVA. Different superscript letters denote significant differences between groups within each sex and fat depot (*P* < 0.05)

**Fig. 4:**
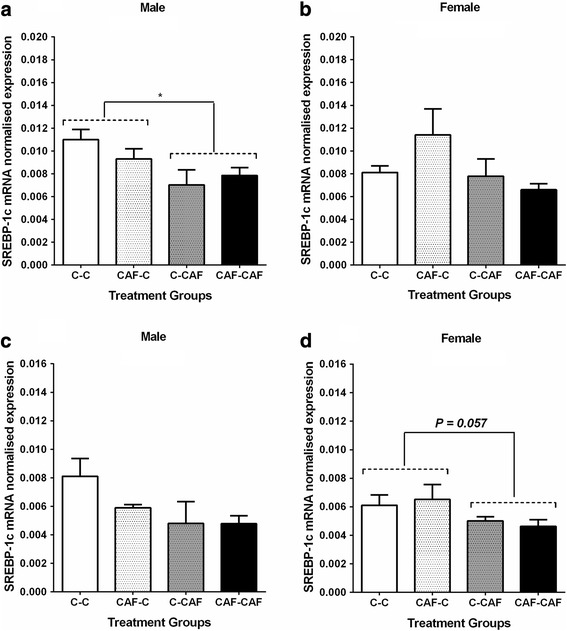
SREBP-1c mRNA expression in male and female offspring in subcutaneous adipose tissue (**a**, **b**) and in retroperitoneal adipose (**c**, **d**) at weaning. Values are expressed as mean ± SEM. * denotes significant differences between treatment groups *(P < 0.05). (C-C, n = 7 males,7 females; CAF-C, n = 4 males, 4 females; C-CAF, n = 4 males, 5 females; CAF-CAF: n = 6 males, 7 females)*

**Fig. 5:**
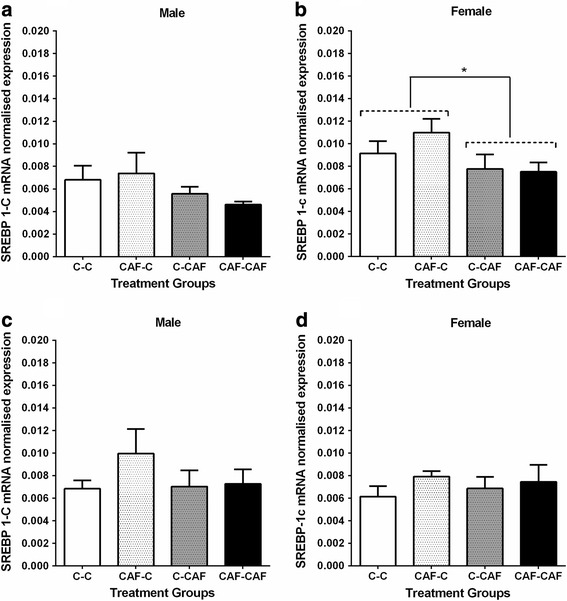
SREBP-1c mRNA expression in male and female offspring in subcutaneous adipose (**a**, **b**) and in retroperitoneal adipose tissue (**c**, **d**) at 6 weeks of age. Values are expressed as mean ± SEM. * denotes significant differences between treatment groups *(P < 0.05) (C-C, n = 8 males, 8 females; CAF-C, n = 4 males, 6 females; C-CAF, n = 4 males, 5 females; CAF-CAF: n = 6 males, 6 females)*

At 3 weeks of age, male offspring suckled by CAF dams had ~ 30% lower expression of SREBP-1c mRNA in the subcutaneous adipose tissue than those suckled by Control dams (Fig. 4a), whilst female offspring suckled by CAF dams tended (*P* < 0.06) to have lower SREBP-1c mRNA in the retroperitoneal adipose depot at 3 weeks (Fig. 4d) and had ~ 25% lower SREBP-1c mRNA expression in subcutaneous adipose tissue at 6 weeks of age (Fig. 5b).

There was no effect of either prenatal or postnatal exposure to the cafeteria diet on G3PDH or FAS mRNA expression in the subcutaneous or retroperitoneal depots at 3 weeks of age in either male or female offspring (Table 6). There was an interaction between the effects of prenatal and postnatal exposure to the cafeteria diet in relation to G3PDH and FAS mRNA expression in the retroperitoneal depot in male offspring at 6 weeks of age, such that those offspring born to CAF dams who were suckled by a Control dam exhibited ~ 2-fold higher expression of both G3PDH and FAS mRNA compared to those born to and suckled by a Control dam (Table 7). There were no effects of either prenatal or postnatal exposure to the cafeteria diet on G3PDH or FAS mRNA expression in the subcutaneous fat depot in males or in either fat depot in females (Table 7).

#### Adiponectin and leptin gene expression in visceral and subcutaneous adipose tissue

At 3 weeks of age, the relative expression of adiponectin and leptin mRNA in the retroperitoneal adipose tissue was higher in both male (2.7 and 3.0 fold, respectively) and female (1.6 and 2.1 fold, respectively) in offspring suckled by CAF dams compared those suckled by Control dams (Table 6). In the subcutaneous adipose tissue, leptin mRNA expression was 2-fold higher in offspring suckled by a CAF dam compared to those suckled by a Control dam, whilst adiponectin mRNA was 1.8-fold higher in female offspring suckled by CAF dams only (Table 6). These effects were independent of whether the offspring had been born to a Control or CAF dam.

At 6 weeks of age, expression of adiponectin and leptin mRNA in the retroperitoneal fat was 1.6 and 2.3-fold lower respectively in female offspring born to CAF dams compared to those born to a Control dam, independent of dietary exposure during the suckling period (Table 7). Adiponectin and leptin mRNA expression in the subcutaneous fat depot was not different between groups in either males or females.

## Discussion

This study provides evidence that exposure to a cafeteria diet during the prenatal or early postnatal period has different effects on fat deposition and the expression of lipogenic/adipokine genes in adipose tissue in the offspring, and that a number of these effects are sex-specific. Our findings suggest that the suckling period plays a more important role in the regulation of both lean tissue growth and fat deposition at weaning than exposure before birth.

### Growth and fat deposition

As reported previously [14, 16], the cafeteria protocol induced significant weight gain in the rat dams prior to and during pregnancy and this was maintained during lactation. The cross-fostering design therefore enabled us to dissect the relative effects of exposure to maternal obesity during pregnancy and lactation. Our finding that offspring suckled by cafeteria dams were lighter at weaning, independent of their dietary exposure before birth, suggests that the nutritional environment during the suckling period plays the more critical role in supporting offspring growth. In addition, while the extent of the weight difference between groups in relative terms was lower at 6 weeks of age, the deficit in body weight was nevertheless maintained in pups suckled by cafeteria diet-fed dams at this time point, even when offspring were fed a nutritionally complete chow post-weaning. The lower body weight of offspring of dams fed a high-fat and/or cafeteria diet during pregnancy and lactation has been reported in a number of previous studies by our group and others [4, 14, 16–20]. This suggests that there are nutritional deficiencies in these diets which result in permanent growth deficits in the offspring. While dams fed the cafeteria diet consumed greater amounts of fat than their control counterparts, it is notable that they consumed ~ 50% less protein during both pregnancy and lactation. While maternal protein restriction is associated with growth restriction in the offspring that persisted until adulthood [21, 22], we have previously reported that the protein content of the milk was not different between dams fed the control and cafeteria diets [23], and it therefore appears unlikely that differences in protein exposure during the suckling period could fully account for the observed growth deficits. The cafeteria diet also contains lower levels of key micronutrients [8], and it is possible that this may contribute to poorer growth of the offspring, even when it occurs in conjunction with a high fat/high-energy density diet. The observations from the present study thus suggest that exposure to a maternal cafeteria diet, low in protein and micronutrient density, during the suckling period alone results in deficits in pup growth which cannot be entirely corrected by providing a nutritionally complete diet after weaning. The suckling period also represents a critical period during which maternal cafeteria feeding impacts upon offspring behaviour, including feeding behaviours [24, 25].

While overall growth was reduced, a key finding of this study was that, independent of whether they were born to a dam consuming a control or cafeteria diet, offspring who were suckled by a dam consuming a cafeteria diet had double the percentage body fat at weaning compared to those suckled by a control dam. This finding is consistent with the cross-fostering study of Sun and colleagues [5] and suggests that the suckling period is a critical window for fat deposition in rodents. Unlike the effects on body weight and linear growth, however, the fat mass of pups suckled by cafeteria dams was normalised after all offspring had consumed a nutritionally balanced chow for 3 weeks after weaning. This suggests that either the post-weaning diet is able to ameliorate the negative effects of exposure to a cafeteria diet earlier in postnatal life on fat deposition, or that whilst the early metabolic trajectory of the offspring favours fat gain, in the absence of nutritional excess there is a normalisation of adiposity.

The absence of any marked persistent metabolic disturbances in these offspring is supported by the lack of any significant effects of exposure to the cafeteria diet during the suckling period on glucose tolerance at 6 weeks of age, and suggests that the adverse effects of early life exposures may not persist into adulthood, at least in the absence of a metabolic challenge. Previous studies, including studies in maternal low protein model, have reported age-dependent changes in glucose-tolerance in the offspring. Thus, low-protein offspring actually have better glucose tolerance than controls in young adulthood (6–12 weeks), but by old adulthood (15–21 months) their glucose tolerance is significantly reduced [26–29]. It is therefore possible that any differences in glucose tolerance between groups in the current study only emerge later in life and/or when offspring are exposed to a metabolic or hormonal challenge, such as pregnancy or a high-fat diet. Alternatively, it is possible that differences in glucose tolerance were present at weaning, when the differences in fat mass/body weight were most pronounced, but were reversed by 3 weeks on the nutritionally balanced diet. It will be of interest in future studies to evaluate insulin signalling genes in muscle, liver and adipose tissue to determine if that is any evidence of alterations to the insulin signalling pathway in offspring exposed to a cafeteria diet before birth or during the suckling period.

The importance of the suckling period for determining both lean tissue/linear growth and fat deposition in the offspring implicates factors within the dam’s milk, the dominant source of nutrition for the offspring during this time, as key contributors. The deficit in lean tissue/linear growth suggests that the milk produced by the cafeteria fed dams contains insufficient levels of key macro/micronutrients and/or growth factors to support optimal neonatal growth and/or that they produce an insufficient volume of milk [30–33]. The changes to breast milk induced by the cafeteria diet also appear to promote fat deposition. We have previously reported that feeding dams a cafeteria diet is associated with a significant increase in total fat content, and the proportion of saturated and trans fatty acids in the breast milk at mid-lactation, with no change in the level of protein [23], however the impact of cafeteria diets on other components of breast milk remains unknown. Studies are needed to further characterise the effects of the cafeteria diet on other nutritional and non-nutritional factors in milk.

### Gene expression

The impact of the cafeteria diet on adipose tissue gene expression was complex, and both sex and depot-specific. The increased mRNA expression of the key adipogenic and lipogenic transcription factor, PPAR-γ mRNA, in subcutaneous adipose observed at weaning in female offspring suckled by cafeteria-fed dams, may have contributed to the increased fat deposition in these offspring, since upregulation of PPAR-γ is known to promote fat storage [34]. Increased expression of PPAR-γ in adipose tissue has also previously been implicated in the programming of increased fat deposition by maternal overnutrition/high-fat feeding in both rodents and sheep [35, 36], and this gene therefore appears to be an important target for early life programming of increased adiposity. It is unclear why the effects on PPAR-γ gene expression did not extend to males in the current study, particularly given that both male and female offspring suckled by cafeteria-fed dams exhibited the same increase in fat mass at weaning. It is possible that the increased fat deposition in male offspring is driven by different underlying mechanisms, potentially altered insulin sensitivity of the adipocyte, or that PPAR-γ regulation occurs at the translational level in males. The increase in PPAR-γ mRNA expression in female offspring suckled by cafeteria-fed dams was no longer observed at 6 weeks of age, suggesting that this effect can be effectively reversed by providing animals with a nutritionally balanced diet after weaning.

The reduced expression of another key lipogenic transcript factor, SREBP-1c, in fat depots of both male and female offspring at 3 weeks suggests that this gene is unlikely to be a target for promoting increased adipose tissue deposition, since downregulation would be expected to reduce, rather than increase, fat deposition [37]. It appears likely, however, that the lower SREBP-1c expression is a consequence of the increased fat deposition (ie. a compensatory mechanism to limit fat storage), in line with previous studies which have shown lower levels of SREBP-1c in obese humans, and suppression of this gene by leptin in mice [38, 39]. While SREBP-1c mRNA levels in male offspring were no longer different between groups at 6 weeks of age, the expression of SREBP-1c mRNA in females was still ~ 25% lower in those offspring suckled by CAF dams. This suggests that the impact of exposure to a cafeteria diet during the major period of adipocyte development in the rodent persists beyond the immediate post-weaning period, even when offspring are consuming a nutritionally-appropriate diet. That this effect was only observed in females is particularly relevant in light of our previous findings in this same animal model, in which female offspring of CAF dams had a greater susceptibility to diet-induced fat deposition when exposed to a highly palatable diet in young adulthood [4]. However, whether this is causally linked to the altered SREBP-1c mRNA expression/regulation remains to be determined.

The increased fat deposition in the offspring suckled by cafeteria dams also did not appear to be driven by an upregulation of the lipogenic genes, G3PDH or FAS, since expression of these genes was not elevated in these offspring at either 3 or 6 weeks of age. Our finding that expression of both G3PDH and FAS was increased by 2-fold in the retroperitoneal adipose tissue of male offspring exposed to a cafeteria diet before birth and a control diet during suckling, at 6 weeks of age, may point to a programming of increased lipogenic capacity of adipose tissue when exposure to the cafeteria diet does not continue after birth. However, our previous studies in this same animal model provide no evidence that this group of male offspring have a greater susceptibility to diet-induced fat deposition [4], and thus the functional significance of these changes remains to be determined. The poor correlation of FAS and SREBP1-c expression is surprising as the latter directly regulates FAS in response to insulin and other signals [27]. This would suggest that effects of the diet on lipogenesis are complex and require further investigation.

Adiponectin mRNA expression at weaning was increased in both male and female offspring suckled by CAF dams compared to controls, and to a greater extent in males compared to females. This was unexpected given that adiponectin concentrations are inversely related to fat mass in adults [40]. However, a number of studies in both animals and humans have reported that plasma adiponectin levels are higher in neonates compared to adults, and are positively, rather than negatively, related to neonatal body weight/fat mass and neonatal weight gain [41, 42]. Thus, adiponectin may potentially be contributing to the increased fat deposition in offspring suckled by CAF dams in the early postnatal period. The increased fat mass in offspring suckled by CAF dams was accompanied by significant increases in both leptin mRNA expression in adipose tissue and circulating plasma leptin concentrations in both males and females. These results are consistent with the well-established role of leptin as a circulating signal of body fat mass in both animals and humans [43]. Interestingly, plasma leptin concentrations were no longer correlated with relative fat mass at 6 weeks of age. This could potentially indicate an altered relationship between fat storage and leptin synthesis in the fat depots of these offspring, however further studies are required to examine this directly. Consistent with a previous study [44], plasma leptin concentrations were also lower at 6 weeks compared to 3 weeks of age, which is likely to be due to the switch from a high-fat milk to a high-carbohydrate diet at weaning.

### Sex differences

In addition to the depot specific outcomes, our findings also suggest that the effect of maternal cafeteria diet exposure on the expression of lipogenic genes is sex-specific. This is in line with previous studies that have reported sex-differences in the programming of lipid metabolism/adipocyte gene expression in response to maternal under/overnutrition [11, 45, 46]. The current study therefore further highlights the need to study both male and female offspring in studies of developmental programming. Further investigations are required to explore the potential interaction between estrogen, other sex-specific hormones and maternal diet in adipose tissue development during the perinatal period.

### Study limitations

A limitation of any study in which experimental diets are provided to animals prior to mating is that it is almost impossible to avoid some variability in the length of time animals are exposed to the diet prior to pregnancy. While this variation in the current was not substantial (all dams were pregnant between 4 and 6 weeks after commencing their experimental diets), there is some evidence to suggest that exposure to high-fat diets for short periods (of just a few days) can result in significant changes in DNA methylation/gene expression, and that these can differ from changes observed following longer periods of exposure to the same diet [47–49]. While we consider that any such differences would be unlikely to have influenced the main conclusions of the study, we cannot completely exclude this possibility, and measurement of both maternal and offspring methylation in similar studies in the future will be of interest.

## Conclusions

We have demonstrated that exposure to a cafeteria diet exclusively during the suckling period is associated with the same magnitude of effects on fat deposition, expression of adipokine genes in the offspring at weaning and reductions in with adipose tissue SREBP-1c mRNA in females at 3 weeks post-weaning, as exposure throughout the entire perinatal period. This finding points to the critical role of factors in the dams milk as in driving fat deposition and, potentially, contributing to programming altered adipocyte function in female offspring, which could in turn contribute to their increased susceptibility to obesity when fed on a cafeteria diet [4]. Therefore, there is a need for further studies to characterise the impact of maternal cafeteria/obesogenic western diets on the full range of nutritional and bioactive components in breast-milk. Our study also provides encouraging data that, at least in male offspring, any long-term effects of exposure to a cafeteria diet during the suckling period on fat deposition and adipocyte gene expression could potentially be mitigated by consuming a nutritionally balanced diet after weaning. While there is a need to exercise caution when extrapolating these findings to a human context, given the different trajectory of development between rodents and humans, this study nevertheless highlights the importance of the lactation period in fat deposition and programming of adipocyte gene expression, and the need to understand more about the impact of different dietary patterns on breast milk composition. Future studies focussed on assessing other aspects of adipocyte metabolism, in particular lipolysis, epigenetic changes and gene expression in other key metabolic organs in our model will be of value for furthering our understanding of the underlying mechanisms.

## Abbreviations

ANOVA: Analysis of variance
AUC: Area under the curve
CAF: Cafeteria diet
CoV: Coefficient of variation
CYPα: Cyclophilin Pα
FAS: Fatty Acid Synthase
G3PDH: Glycerol-3-phosphate dehydrogenase
IPGTT: Intraperitoneal glucose tolerance test
PPAR-γ: Peroxisome Proliferator Activated Receptor-γ
qRT PCR: Quantitative real time PCR
SEM: Standard error of the mean
SREBP-1c: Sterol Regulatory Element Binding Protein-1c
